# Supplementary material for: Causal graph analysis of COVID-19 observational data in German districts reveals effects of determining factors on reported case numbers
Source: PLoS One. 2021 May 27;16(5):e0237277. doi: 10.1371/journal.pone.0237277 (PMC8158986; doi:10.1371/journal.pone.0237277)
Supplement: Attachment — Submitted filename: replyreviewer.pdf [file pone.0237277.s001.pdf]

Reply to Reviewer

We want to thank the reviewer for the feedback. We heavily revised the manuscript to address every remark of the reviewer. Below we will address every point of the review separately.

This is an interesting manuscript that aims to identify "causal" relationships" for COVID-19 transmission based on a DAG analysis and empirical data from other studies for variable selection.

1. Yes, we aim to estimate *causal effects* based on a DAG that was constructed based on other publications. Empirical data was extracted and aggregated from different publicly available databases: RKI, Google mobility/Trends, INKAR, DWD. Variable selection for the different models was based on the proposed DAG using the well-studied methods from Pearl, Spirtes, and others. That is, rather than selecting variables, we identified valid adjustment sets in a principled way to estimate effects in a causal framework.

The DAG analysis is a multivariate analysis to select a subset of variables that are correlated from a large number of candidates. The first select the sets of variables that are associated with the outcome using DAG analysis and then put them into the regression. The DAG analysis returns different sets of variables, using different selection criterion (for example, the most parsimonious set or the most variation explained). So the authors assumedly use R-squared to select the final sets of variables in the regression model.

2. Yes, for every exposure variable separately, we identify subsets of variables to include in the regression models using DAG analysis. We stress the point that we do this identification process separately for every variable of interest where we are interested in the total causal effect. We always regress on the same outcome Y (Reported COVID-19 cases) using negative binomial regression. That is, we select one variable Xi where we are interested in the effect of Xi on Y. Then we identify all possible subsets of variables in the DAG that allow to estimate the causal effect of Xi on Y (based on the do-calculus framework by Pearl et al.). Out of these subsets we choose the "best" one in regards to minimal AIC (we changed this from R-squared in the new version of the manuscript). We repeat this procedure for all possible exposure variables Xi. We want to stress the point, that the set of valid adjustment sets is not found by looking at AIC/R-squared (or the actual data), but rather only the structure of the DAG. These adjustment sets have the property to allow reliable estimates of the total causal effect from Xi to Y. When given multiple adjustment sets for the same Xi, we now chose the one with the minimal AIC.

To sum it up, our strategy aims to identify for every exposure variable separately the adjustment set with the best statistical properties among all valid adjustment sets.

The challenge I have here (and having reviewed this as well with our statistical team) is that the claims of causal relationships are not convincing, even if they identify associative relationships that other studies have also found.

3. We are aware that the causal DAG framework is still a rather new approach to study relationships between different factors. It is fascinating how the causal DAG and do-calculus framework allows to actually discern mere associations from actual (direct or total) causal effects. Of course, this is only possible if the DAG is reliable and regression model assumptions are met. We are happy to discuss the proposed DAG. To reflect the concerns that we actually found *causal* effects, we used more careful

wording throughout most of the revised paper, to make clear that effects estimates are based on the causal graph and are strong indicators for causal relationships. Within the causal DAG framework, the terms *total/direct causal effect* are used to describe the relationships (that is, arrows) between variables/nodes in the causal DAG.

Specifically: 1) The model does not address the nonlinearity of variables (such as temperature); other studies have found that temperature has a u-shaped effect at low and high temperatures. Humidity effects may also be nonlinear. The assumption of non-linearity on continuous variables needs to be considered.

4. Yes, non-linearity might be a problem for our generalized linear model (negative binomial regression). We did a complete check of model diagnostics that addresses linearity assumptions, model specification/distribution of residuals, independence of observations, and multi-collinearity checks (see below). We did not find significant problems with non-linear relationships.

The fitted vs residual plot shows general good model adequacy, since the residuals are almost evenly distributed around 0 for all fitted values. This means that the negative binomial model is adequate and there is no general non-linear trend in the predictors collectively (otherwise the black line would show a clear trend down/up/strongly curved).

The plot for individual predictors versus residuals does not reveal strong non-linear effects for individual predictors. The LOESS curves for COVID-19 burden and Mobility Principal Component 3 wiggle upwards for higher values, but these are very few. In the majority, curves are horizontal and don't imply model problems with non-linearity.

The distribution of the quantile residuals is a very good fit to a normal distribution, which indicates that the negative binomial model is a good model for the data. (For generalized linear models, QQ-plots of Pearson residuals are not meaningful, since a GLM actually makes no assumption about the distribution of errors.)

We did not model "time" explicitly as a variable. The residual plot with residuals ordered by time shows that we can assume independence of observations over time within the model, since the residuals are independent and identically distributed (iid) around 0 for every date. In reality, observations on subsequent days are not independent, but the data does not show a strong temporal trend in residuals.

Another viewpoint on independence of observations: Since our data comes from 401 different districts, a mixed/hierarchical model might be more appropriate, since we cannot assume that observations within the same district are independent of each other. The plot of district id vs residuals doesn't show a strong violation of the independence assumption (we actually ordered the districts by their respective residual mean in the plot above). Nevertheless, we tried a mixed model with random intercepts for districts, but the very small increase in model performance was in no relation to the increased computing time by several magnitudes.

Assessing these diagnostic plots, we are confident that our negative binomial regression framework is appropriate and that non-linearity is not an issue with this dataset.

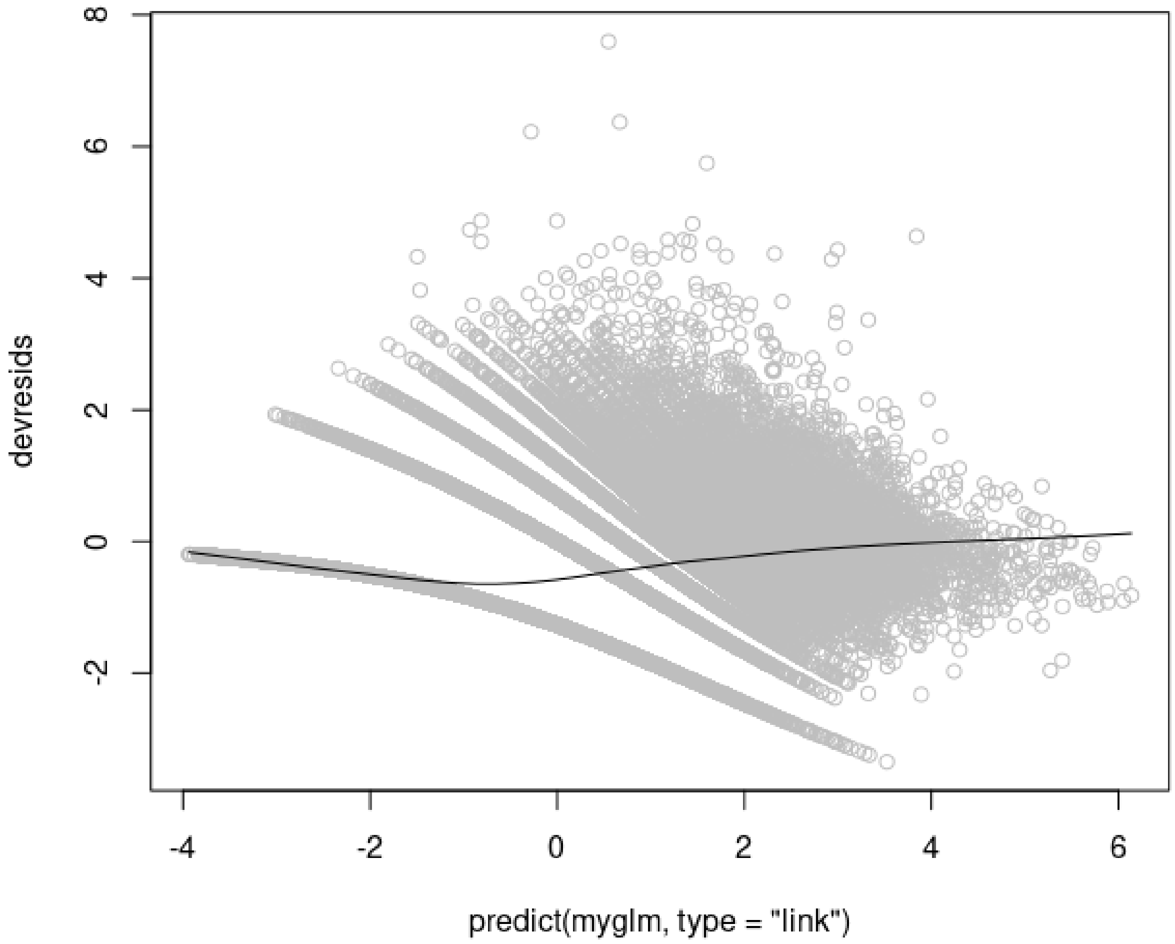

Figure 1: Diagnostic plot: Fitted vs residuals.

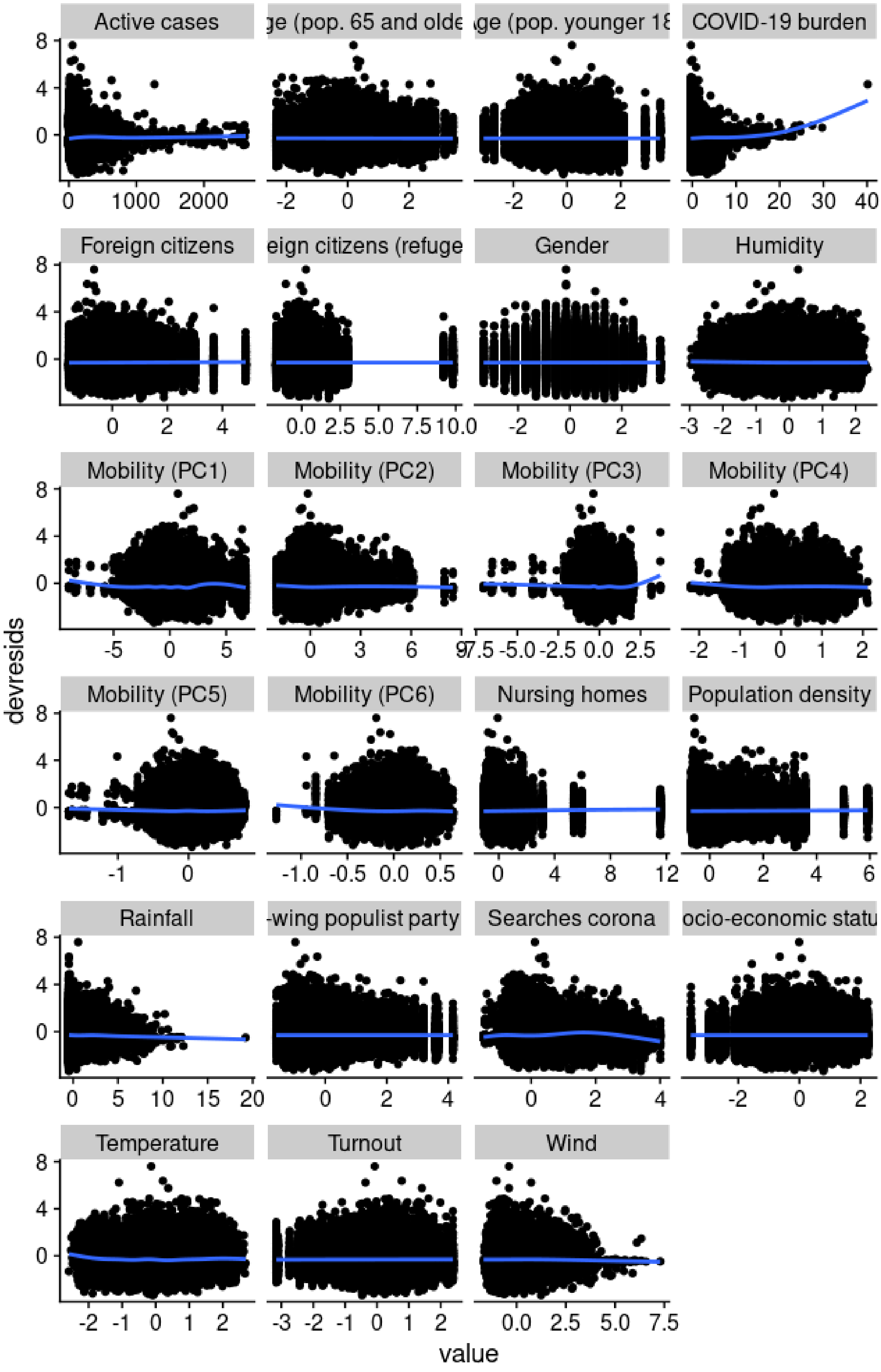

Figure 2: Diagnostic plot: predictors vs residuals.

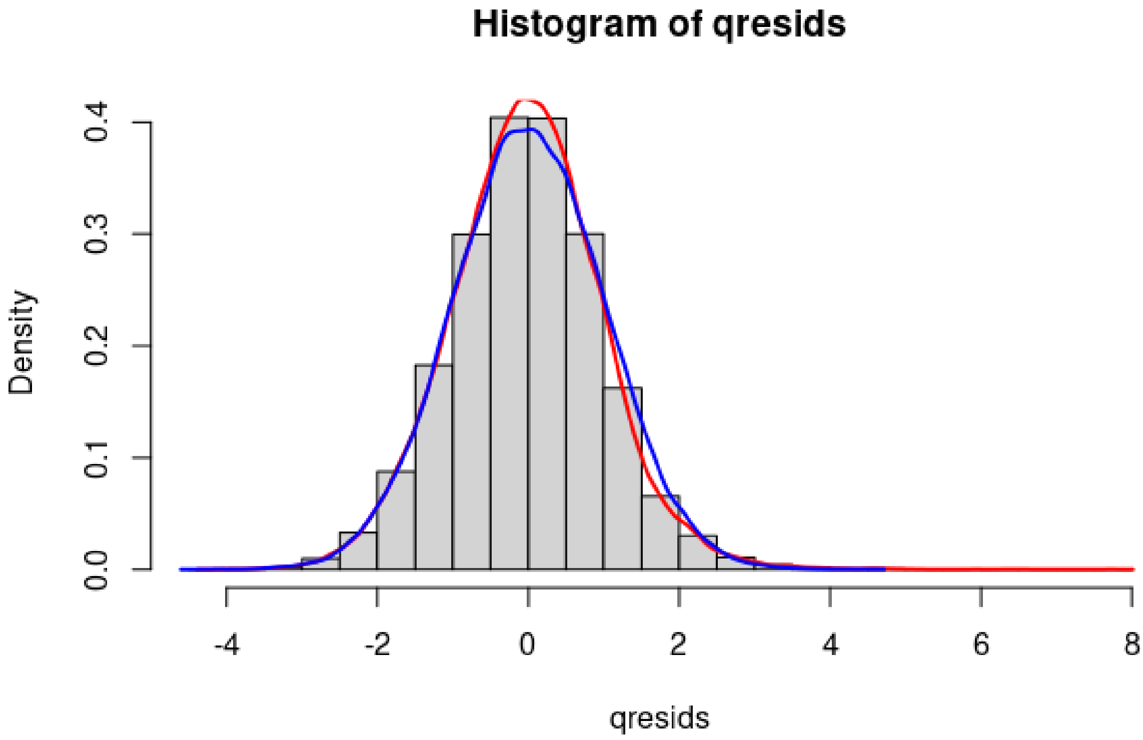

Figure 3: Diagnostic plot: distribution of residuals.

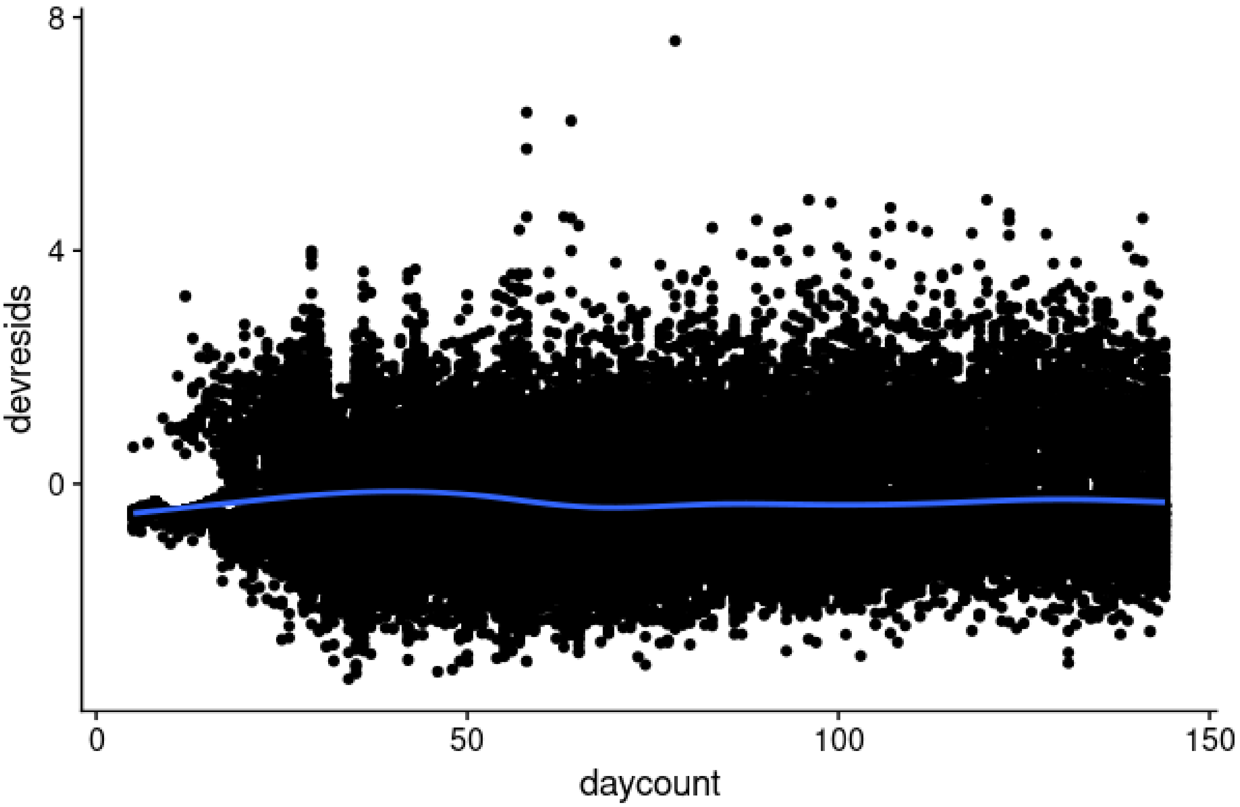

Figure 4: Diagnostic plots: independence of observations, temporal.

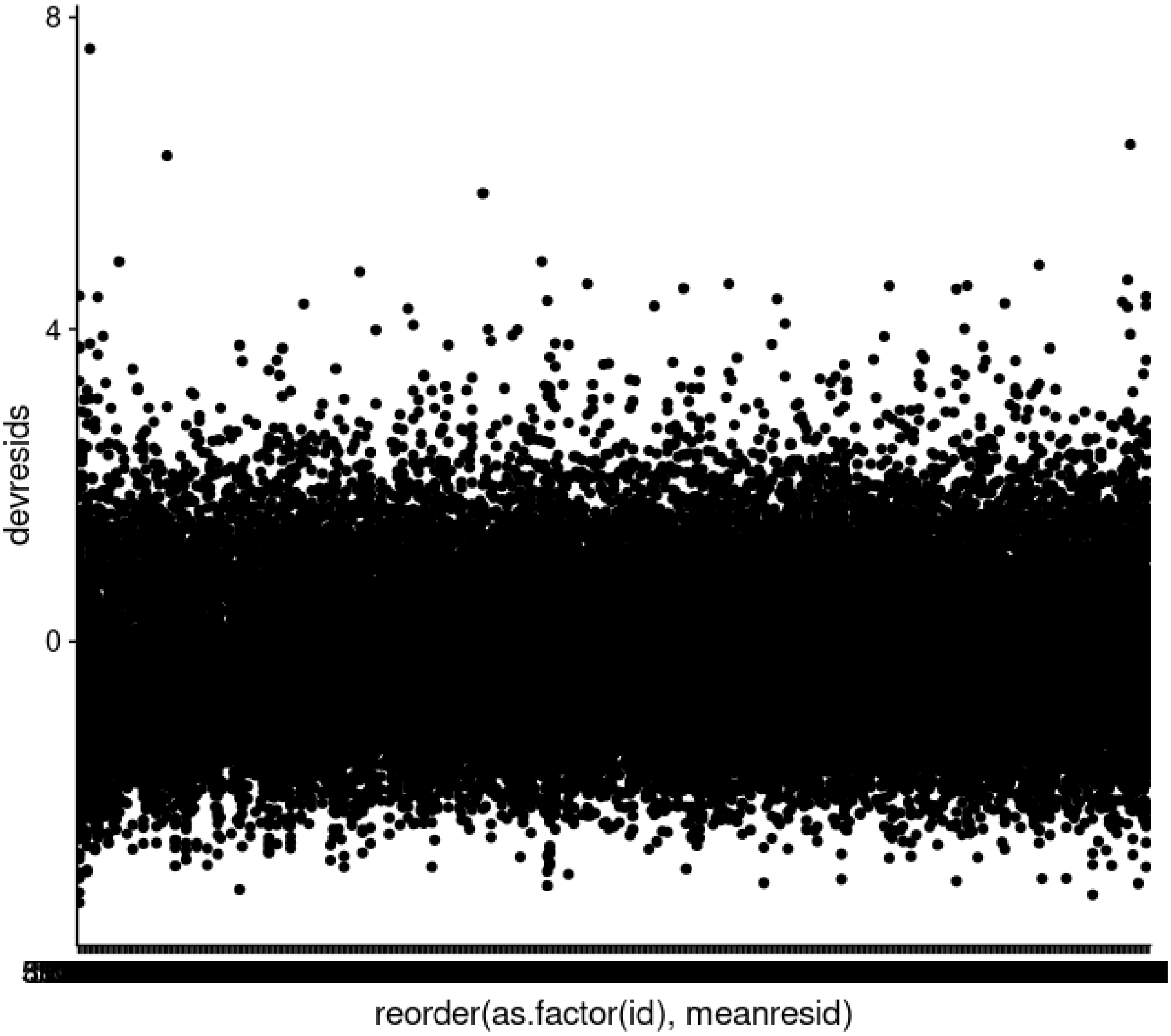
8
4
0
devresids
reorder(as.factor(id), meanresid)

2) There are no tests for multi-collinearity that are presented, which presents significant concerns for over-fitting the model. Temperature and humidity are an example here. So is the mobility data.

5. This an important remark which led to some major revisions on the analysis pipeline and the paper. We assessed variance inflation factors (VIFs) for the full model. We observed high VIFs for mobility variables and intervention variables. We decided to transform the original mobility variables by PCA and used the set of these principal components instead of the original mobility variables. Furthermore, we employed ridge regression as a way to further deal with multicollinearity and overfitting. We want to add that causal graph analysis with regression is more robust to multicollinearity than traditional regression approaches (see Schisterman et al 2017), and that in the presence of correlated variables, one might miss important effects, but the effects found are still valid. To find the most appropriate hyper-parameter for the ridge regression, we employed cross-validation on subsets of the 401 districts. Due to employing ridge regression for the effect estimates, we cannot report meaningful confidence intervals/p-values. Furthermore, we used cross-validation for identifying the most appropriate lag parameter, which was subsequently changed from 8 to 5 days in the manuscript.

2) Feels like they cherry-pick the variables of interest in the end? Not sure how they arrived at the final variable list for causal effects. Whey were the restrictions not included in that analysis?

6. At the time of writing, we were more interested in variables that could be prognostive of future developments (especially weather, since weather is not modifiable). In light of this remark, we revised the analysis and paper and now we include all variables from the DAG as possible exposures, and we report and discuss all effect estimates in the manuscript. To reflect this, we changed the title of the manuscript, too.

3) The term "causal analysis" is a bit strong for what they have done here. The basis of the work is the proposed DAG (Figure 1), but the diagram was constructed from other association studies. Other than the DAG analysis, they did not do anything to ensure the results are "causal relationship". So I am not convinced that the analysis or results are causal.How do you avoid over-fitting the model or including mediating variables in the analysis?

7. Please see our remarks 1., 2., and 3. that address the general idea of/concerns about causal frameworks with DAGs and do-calculus. We use the term "causal" the same way that Pearl (Causality: Models, Reasoning, and Inference), Spirtes et al. (Causation, Prediction, and Search) and the growing body of work based on causal analysis with DAGs use it. We modified the title of our paper to "Causal graph analysis of [. . .]" to reflect the fundamental assumption of a causal graph to be able to do causal analysis.

Proper identification of valid variable subsets for causal effect estimation takes care of the mediating variable problem, since this is at the heart of causal DAG analysis.

Overfitting might still be a problem, which we addressed by regularized regression via ridge regression (see above) and updating our analysis, estimates, and the manuscript accordingly.

5) What about interaction terms? For example, residential mobility and colder weather? Or rain? These relationships are not simply a straightforward multivariable model.

8. DAG analysis explicitly takes care of interaction between variables by identifying valid subsets of variables that need to be included or excluded in the multivariable model. We added a remark on interaction to the paper to reflect on this remark.

6) The most interesting terms in their model were the interventions (restrictions) themselves, yet they were dropped from the model. No discussion is really pursued on that. Why were public health interventions removed?

9. See our remark 6., we added effect estimates and discussion of effects for all variables to the manuscript.

Final remarks: We sincerely hope that our remarks, explanations, and the major revisions we did on the analysis and manuscript address all concerns. We included all possible variables as exposure, did a model diagnostics, and added principal component analysis, ridge regression, and cross-validation to the analysis to make it resilient to multicollinearity and overfitting. We hope to provide a meaningful addition to the growing body of research on causal graphs/causal analysis in epidemiology, while reporting important results on the determining factors of the COVID-19 pandemic.
