# Supplementary material for: Causal graph analysis of COVID-19 observational data in German districts reveals effects of determining factors on reported case numbers
Source: PLoS One. 2021 May 27;16(5):e0237277. doi: 10.1371/journal.pone.0237277 (PMC8158986; doi:10.1371/journal.pone.0237277)
Supplement: Attachment — Submitted filename: replyreviewer.pdf [file pone.0237277.s002.pdf]

Reply to Reviewer #2 / Journal Requirements

We want to thank the reviewer for the feedback. We revised the manuscript to address the remark of the reviewer regarding social determinants of COVID-19 infections:

I find the topic of the paper very important and the methodology very interesting. The author has used a number of variables to analyse possible determinants of COVID-19 infections. While the paper acknowledges that there are social factors which affect the spread of the COVID-19 this is not properly discussed in the paper. I strongly suggest the author to refer to the literature on Social Determinants of Health when discussing social variables. For example see the recent paper by Galanis and Hanieh in Social Science and Medicine on Incorporating Social Determinants of Health into Modelling of COVID-19 and references therein.

We extended the Discussion section of our manuscript to address this rightful remark. More specifically, we included 3 additional references to reflect on the situation of social determinants on COVID-19 infections especially in Germany, most prominently the socio-economic status of households.

Furthermore we reply to the Journal Requirements regarding references within the manuscript:

Please review your reference list to ensure that it is complete and correct. If you have cited papers that have been retracted, please include the rationale for doing so in the manuscript text, or remove these references and replace them with relevant current references. Any changes to the reference list should be mentioned in the rebuttal letter that accompanies your revised manuscript. If you need to cite a retracted article, indicate the article's retracted status in the References list and also include a citation and full reference for the retraction notice.

We checked all references, as of 19 April 2021 none were retracted since the first version of the manuscript. We updated some references that moved from a pre-print status to being published after peer-review. Furthermore, we cleaned up the reference list from faulty ISSN.
